# Two species of the genus *Valenzuela* (Psocodea, Psocomorpha, Caeciliusidae), newly recorded from South Korea

**DOI:** 10.3897/BDJ.14.e216503

**Published:** 2026-10-23

**Authors:** Jaeyun Kim, Wonhoon Lee

**Affiliations:** 1 Department of Plant Medicine, Gyeongsang National University, Jinju, Republic of Korea Department of Plant Medicine, Gyeongsang National University Jinju Republic of Korea; 2 Institute of Agriculture & Life Science, Gyeongsang National University, Jinju, Republic of Korea Institute of Agriculture & Life Science, Gyeongsang National University Jinju Republic of Korea

**Keywords:** Caeciliusidae, faunistics, identification key, new records, Psocodea, Psocomorpha, South Korea, *
Valenzuela
*

## Abstract

**Background:**

The genus *Valenzuela* Navás, 1924 (Psocodea, Psocomorpha, Caeciliusidae) currently comprises 297 extant species worldwide. However, only one species, *Valenzuela oyamai* (Enderlein, 1906), has previously been recorded from South Korea. Further examination of specimens collected in the country is needed to improve knowledge of the national fauna and provide morphological references for species identification. In this study, psocid specimens collected from several localities in South Korea between 2023 and 2025 were examined to document additional species of *Valenzuela* and their diagnostic characters.

**New information:**

*Valenzuela badiostigma* (Okamoto, 1910) and *Valenzuela flavidus* (Stephens, 1835) are recorded from South Korea for the first time, increasing the number of *Valenzuela* species known from the country from one to three. Descriptions and measurements, based on Korean specimens, are provided, together with illustrations of diagnostic characters and information on collection localities and geographical distribution. An identification key to the three *Valenzuela* species currently known from South Korea is also presented. These records update the national inventory of the genus and provide reference material for subsequent taxonomic studies and faunistic surveys.

## Introduction

Free-living psocids, commonly known as barklice and booklice, are small insects that include species, feeding on fungi, algae, lichens and organic debris. Despite their taxonomic diversity, these inconspicuous insects have often received limited attention in faunistic research (Soysouvanh et al. 2017). Caeciliusidae is a family within the infraorder Caeciliusetae of the suborder Psocomorpha (Psocodea). According to the Psocodea Species File, the family currently comprises 36 genera and 644 extant species worldwide (Hopkins et al. 2026).

Within Caeciliusidae, the genus *Valenzuela* Navás, 1924 comprises 297 extant species, accounting for nearly half of the extant species currently assigned to the family (Hopkins et al. 2026). For a species-rich genus, regional taxonomic accounts provide an important link between global catalogues and locally collected material. In particular, occurrence records accompanied by descriptions, measurements and illustrations allow the morphological basis of identifications to be evaluated. Such documentation also provides a reference for comparing subsequently collected specimens and assessing the species composition of regional faunas.

In South Korea, Soysouvanh et al. (2017) reviewed museum collections and recently collected specimens and reported ten additional species of psocids from the country. Their study included the first Korean record of *V. oyamai* (Enderlein, 1906), a species also reported from Japan, China, Taiwan and the Russian Far East. Before the present study, *V. oyamai* was the only species of *Valenzuela* recorded from South Korea (Soysouvanh et al. 2017). Further specimen-based investigations are, therefore, needed to improve the national inventory of this genus. Documenting additional occurrences and their diagnostic characters would also provide a firmer basis for future comparisons with other East Asian faunas.

Examination of psocid specimens collected from several localities in South Korea between 2023 and 2025 revealed two species previously unrecorded from the country: *V. badiostigma* (Okamoto, 1910) and *V. flavidus* (Stephens, 1835). Here, we document their first South Korean records and provide descriptions, measurements and illustrations of diagnostic characters, based on the examined specimens, together with information on their collection localities and geographical distribution. We also present an identification key to the three *Valenzuela* species now known from South Korea. By combining occurrence data with morphological documentation, this study provides a reference for the identification of Korean *Valenzuela* and a foundation for further faunistic investigation.

## Materials and methods

Each specimen was individually preserved in a microtube containing 99% ethanol and stored at −20°C. The general morphology of the body, head and wings was examined using a Leica M205C stereomicroscope. The right wings were dissected for detailed morphological examination. To examine the genitalia, the abdomen was dissected and cleared in 10% potassium hydroxide (KOH) for one hour. The genital structures were stained with Wilkey’s double stain before mounting. The dissected wings and genitalia were mounted in Canada balsam and examined using a Leica DM6B microscope.

Species identification was based on the relevant taxonomic literature (Tomita and Haga 1991, Lienhard 1977, Mockford 2000, Yoshizawa 2005).

Terminology for adult morphology, wing venation and terminalia follows published conventions (Yoshizawa 2005).

All examined specimens are deposited in the Insect Collection of Gyeongsang National University, Jinju, Republic of Korea (GNU).

Abbreviations of parts measured are as follows: FW, HW: lengths of right fore- and hind-wings; F, T, t1 and t2: lengths of femur, tibia and tarsomeres 1 and 2 of right hind leg, respectively; f1–f2: lengths of flagellomeres 1–2 of right antenna; dv: dorsal valve; ev: external valve.

## Taxon treatments

### Valenzuela badiostigma

(Okamoto, 1910)

CC61FA8C-7868-58CD-B2B1-198FE255646E

Caecilius badiostigma Okamoto (1910): 206.
Valenzuela badiostigma (Okamoto, 1910): Mockford (2000): 351.

#### Materials

- **Type status:** Other material. **Occurrence:** catalogNumber: coll#.JY-230918; recordedBy: J. Kim; individualCount: 1; sex: female; lifeStage: adult; occurrenceStatus: present; occurrenceID: 497FAC27-A822-540A-BC89-EE3F0CA7110A; **Taxon:** scientificName: Valenzuela badiostigma; kingdom: Animal; phylum: Arthropoda; class: Insecta; order: Psocodea; family: Caeciliusidae; genus: Valenzuela; taxonomicStatus: accepted; **Location:** country: South Korea; countryCode: KR; stateProvince: Gangwon-do; municipality: Pyeongchang-gun; locality: Daegwallyeong-myeon; verbatimLatitude: 37.403180; verbatimLongitude: 128.422772; geodeticDatum: WGS84; georeferenceProtocol: label; **Identification:** identifiedBy: J. Kim; dateIdentified: 2023; **Event:** samplingProtocol: hand collected; year: 2023; month: 9; day: 18; **Record Level:** type: specimen; language: en; rightsHolder: Gyeongsang National University; collectionID: GNU; collectionCode: Insects; basisOfRecord: PreservedSpecimen
- **Type status:** Other material. **Occurrence:** catalogNumber: coll#.JY-230924; recordedBy: J. Kim; individualCount: 2; sex: male; lifeStage: adult; occurrenceStatus: present; occurrenceID: 9DDA19E7-ACA3-50AF-9B25-BF1EF77E925F; **Taxon:** scientificName: Valenzuela badiostigma; kingdom: Animal; phylum: Arthropoda; class: Insecta; order: Psocodea; family: Caeciliusidae; genus: Valenzuela; taxonomicStatus: accepted; **Location:** country: South Korea; countryCode: KR; stateProvince: Gangwon-do; municipality: Wonju-si; locality: Heungeop-myeon; verbatimLatitude: 37.172256; verbatimLongitude: 127.545940; geodeticDatum: WGS84; georeferenceProtocol: label; **Identification:** identifiedBy: J. Kim; dateIdentified: 2023; **Event:** samplingProtocol: hand collected; year: 2023; month: 9; day: 24; **Record Level:** type: specimen; language: en; rightsHolder: Gyeongsang National University; collectionID: GNU; collectionCode: Insects; basisOfRecord: PreservedSpecimen
- **Type status:** Other material. **Occurrence:** catalogNumber: coll#.JY-241003; recordedBy: J. Kim; individualCount: 1; sex: female; lifeStage: adult; occurrenceStatus: present; occurrenceID: A4EA6B85-0BBB-54A7-BF93-A7019B5E30C9; **Taxon:** scientificName: Valenzuela badiostigma; kingdom: Animal; phylum: Arthropoda; class: Insecta; order: Psocodea; family: Caeciliusidae; genus: Valenzuela; taxonomicStatus: accepted; **Location:** country: South Korea; countryCode: KR; stateProvince: Chungcheongnam-do; municipality: Taean-gun; locality: Anmyeon-eup; verbatimLatitude: 36.312172; verbatimLongitude: 126.213096; geodeticDatum: WGS84; georeferenceProtocol: label; **Identification:** identifiedBy: J. Kim; dateIdentified: 2024; **Event:** samplingProtocol: hand collected; year: 2024; month: 10; day: 3; **Record Level:** type: specimen; language: en; rightsHolder: Gyeongsang National University; collectionID: GNU; collectionCode: Insects; basisOfRecord: PreservedSpecimen
- **Type status:** Other material. **Occurrence:** catalogNumber: coll#.JY-241008; recordedBy: J. Kim; individualCount: 1; sex: male; lifeStage: adult; occurrenceStatus: present; occurrenceID: 73E01A5D-2D41-5508-A359-4001DFC41FD9; **Taxon:** scientificName: Valenzuela badiostigma; kingdom: Animal; phylum: Arthropoda; class: Insecta; order: Psocodea; family: Caeciliusidae; genus: Valenzuela; taxonomicStatus: accepted; **Location:** country: South Korea; countryCode: KR; stateProvince: Gyeongsangbuk-do; municipality: Sangju-si; locality: Namjang-dong; verbatimLatitude: 36.250692; verbatimLongitude: 128.065288; geodeticDatum: WGS84; georeferenceProtocol: label; **Identification:** identifiedBy: J. Kim; dateIdentified: 2024; **Event:** samplingProtocol: hand collected; year: 2024; month: 10; day: 8; **Record Level:** type: specimen; language: en; rightsHolder: Gyeongsang National University; collectionID: GNU; collectionCode: Insects; basisOfRecord: PreservedSpecimen
- **Type status:** Other material. **Occurrence:** catalogNumber: coll#.JY-241016; recordedBy: J. Kim; individualCount: 2; sex: female; lifeStage: adult; occurrenceStatus: present; occurrenceID: C0B82710-82DB-547B-862D-35C1471E9005; **Taxon:** scientificName: Valenzuela badiostigma; kingdom: Animal; phylum: Arthropoda; class: Insecta; order: Psocodea; family: Caeciliusidae; genus: Valenzuela; taxonomicStatus: accepted; **Location:** country: South Korea; countryCode: KR; stateProvince: Gyeongsangbuk-do; municipality: Uiseong-gun; locality: Geumseong-myeon; verbatimLatitude: 36.155076; verbatimLongitude: 128.412220; geodeticDatum: WGS84; georeferenceProtocol: label; **Identification:** identifiedBy: J. Kim; dateIdentified: 2024; **Event:** samplingProtocol: hand collected; year: 2024; month: 10; day: 16; **Record Level:** type: specimen; language: en; rightsHolder: Gyeongsang National University; collectionID: GNU; collectionCode: Insects; basisOfRecord: PreservedSpecimen
- **Type status:** Other material. **Occurrence:** catalogNumber: coll#.JY-241022; recordedBy: J. Kim; individualCount: 2; sex: female; lifeStage: adult; occurrenceStatus: present; occurrenceID: 3530887E-FEEC-5FC5-AA28-A4DEC5E292F9; **Taxon:** scientificName: Valenzuela badiostigma; kingdom: Animal; phylum: Arthropoda; class: Insecta; order: Psocodea; family: Caeciliusidae; genus: Valenzuela; taxonomicStatus: accepted; **Location:** country: South Korea; countryCode: KR; stateProvince: Gyeongsangbuk-do; municipality: Cheongsong-gun; locality: Juwangsan-myeon; verbatimLatitude: 36.212412; verbatimLongitude: 129.090216; geodeticDatum: WGS84; georeferenceProtocol: label; **Identification:** identifiedBy: J. Kim; dateIdentified: 2024; **Event:** samplingProtocol: hand collected; year: 2024; month: 10; day: 22; **Record Level:** type: specimen; language: en; rightsHolder: Gyeongsang National University; collectionID: GNU; collectionCode: Insects; basisOfRecord: PreservedSpecimen
- **Type status:** Other material. **Occurrence:** catalogNumber: coll#.JY-250904; recordedBy: J. Kim; individualCount: 2; sex: female; lifeStage: adult; occurrenceStatus: present; occurrenceID: 2F2C936B-557F-55CF-BF9C-B97692944478; **Taxon:** scientificName: Valenzuela badiostigma; kingdom: Animal; phylum: Arthropoda; class: Insecta; order: Psocodea; family: Caeciliusidae; genus: Valenzuela; taxonomicStatus: accepted; **Location:** country: South Korea; countryCode: KR; stateProvince: Gyeongsangnam-do; municipality: Jinju-si; locality: Gajwa-dong; verbatimLatitude: 35.091771; verbatimLongitude: 128.063607; geodeticDatum: WGS84; georeferenceProtocol: label; **Identification:** identifiedBy: J. Kim; dateIdentified: 2025; **Event:** samplingProtocol: hand collected; year: 2025; month: 9; day: 4; **Record Level:** type: specimen; language: en; rightsHolder: Gyeongsang National University; collectionID: GNU; collectionCode: Insects; basisOfRecord: PreservedSpecimen
- **Type status:** Other material. **Occurrence:** catalogNumber: coll#.JY-250911; recordedBy: J. Kim; individualCount: 2; sex: male; lifeStage: adult; occurrenceStatus: present; occurrenceID: 7958DB2D-485E-57B7-AA9D-22079A59D28A; **Taxon:** scientificName: Valenzuela badiostigma; kingdom: Animal; phylum: Arthropoda; class: Insecta; order: Psocodea; family: Caeciliusidae; genus: Valenzuela; taxonomicStatus: accepted; **Location:** country: South Korea; countryCode: KR; stateProvince: Gyeongsangnam-do; municipality: Geoje-si; locality: Geoje-si; verbatimLatitude: 34.491704; verbatimLongitude: 128.413912; geodeticDatum: WGS84; georeferenceProtocol: label; **Identification:** identifiedBy: J. Kim; dateIdentified: 2025; **Event:** samplingProtocol: hand collected; year: 2025; month: 9; day: 11; **Record Level:** type: specimen; language: en; rightsHolder: Gyeongsang National University; collectionID: GNU; collectionCode: Insects; basisOfRecord: PreservedSpecimen

#### Description

**Figure 1.**
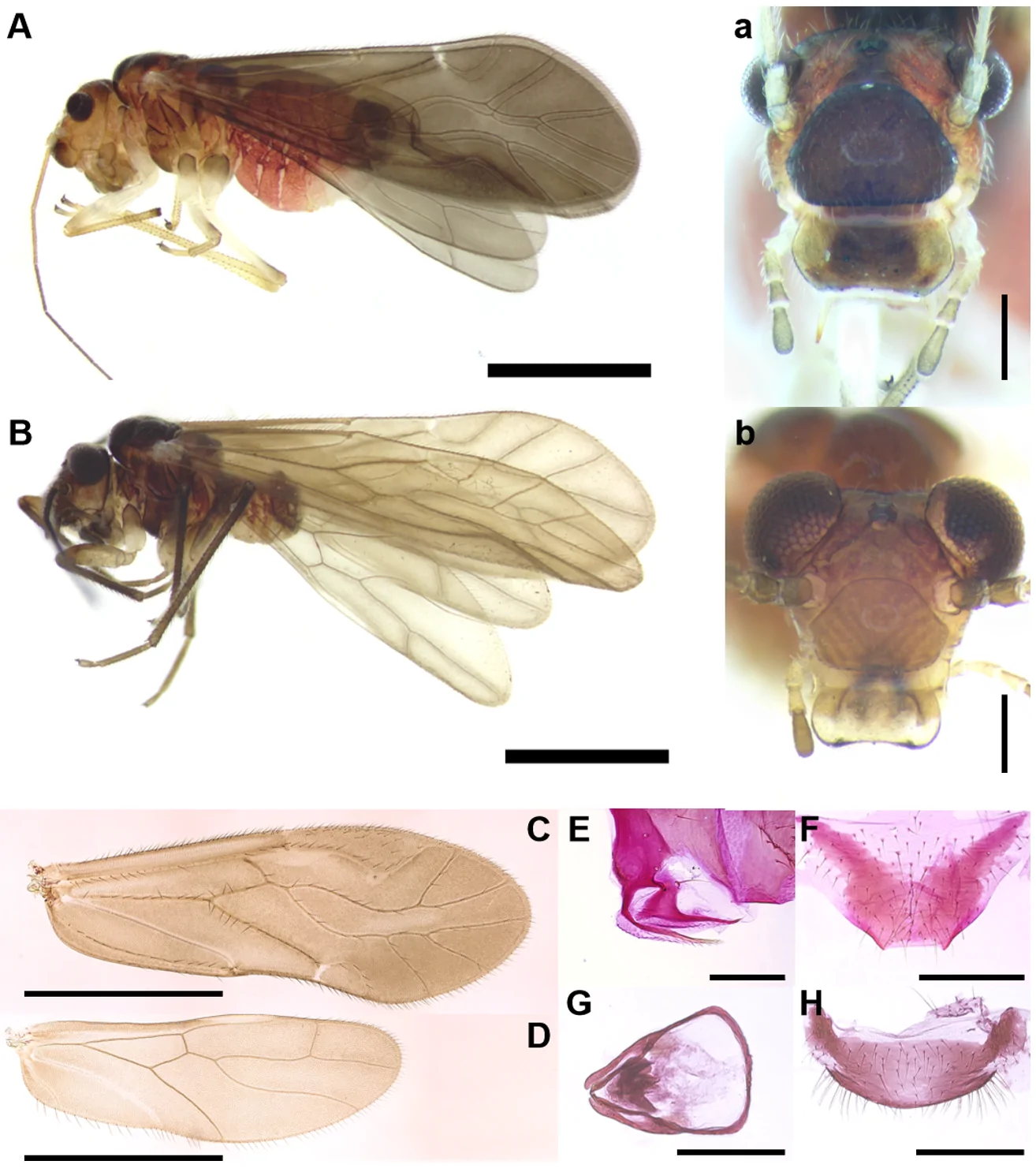
*Valenzuela badiostigma* (Okamoto, 1910). **A** female habitus; a, female head; **B** male habitus; b, male head; **C** fore-wing; **D** hind-wing; **E** gonapophyses; **F** subgenital plate; **G** phallosome; **H** hypandrium. Scale bars: A–D = 1 mm; a, b, F, H = 200 μm; E, G = 100 μm.

**Female**. Body generally dark brown to reddish-brown. Thorax dark brown, nearly black; legs pale yellowish-brown. Abdomen pink dorsally and ventrally, darkening to red laterally (Fig. 1A). Head reddish-brown with darker markings on vertex, frons and ocellar region; eyes black (Fig. 1A). Antenna brown to dark brown, with the basal flagellomeres paler (Fig. 1A). Fore-wing brown, darker posterior to vertex of pterostigma, always a distinct hyaline area near base of pterostigma; often an elongate hyaline window between r and m (Fig. 1C). Hind-wing paler brown than fore-wing, veins brown, narrowly bordered hyaline, except CuP and A widely so (Fig. 1D). Subgenital plate with two distinct apical lobes (Fig. 1F). Gonapophyses with ev reduced and represented only by setae; median portion of dv broadly developed and trapezoidal in shape (Fig. 1E). **Male**. Similar to female in general colouration (Fig. 1B), but eyes larger (Fig. 1B). Antenna and legs mostly dark brown (Fig. 1B). Hypandrium simple (Fig. 1H); phallosome relatively small (Fig. 1G).

#### Distribution

Palaearctic Region (Japan, South Korea [in this study]); Oriental Region (Indonesia); Oceanian Region (Hawaiian Islands) (Lienhard and Smithers 2002).

#### Notes

The examined specimens were collected from foliage and branches of broad-leaved trees and ornamental shrubs, including *Prunus* spp., *Ligustrum* spp. and *Zelkova serrata*. This species is characterised by its generally dark body colouration and fore-wings with translucent spots. The present records suggest that *V. badiostigma* occurs widely from inland regions to southern coastal areas of South Korea.

#### Measurements (mm)

**Female** (n = 8). FW 2.85–3.25; HW 2.05–2.32; F 0.61–0.72; T 1.05–1.23; t1 0.35–0.42; t2 0.12–0.14; f1 0.48–0.57; f2 0.39–0.47. **Male** (n = 5). FW 2.70–3.05; HW 2.05–2.28; F 0.58–0.66; T 0.98–1.14; t1 0.33–0.39; t2 0.11–0.13; f1 0.45–0.53; f2 0.36–0.43.

### Valenzuela flavidus

(Stephens, 1835)

617B1AA4-9E60-5092-823A-57970A6CC6DC

Psocus flavidus Stephens (1835): 122.
Psocus ochropterus Stephens (1835): 122.
Psocus flavicans Stephens (1835): 123.
Psocus subpunctatus Stephens (1835): 126.
Caecilius strigosus Curtis (1837):648.
Psocus boreellus Zetterstedt (1840): 1053.
Psocus striatus Zetterstedt (1840): 1053.
Psocus aurantiacus Hagen 1861: 14.
Caecilius flavidus (Stephens, 1835): Hagen (1866): 205.
Valenzuela flavidus (Stephens, 1835): Janetschek (1949): 114.

#### Materials

- **Type status:** Other material. **Occurrence:** catalogNumber: coll#.JY-230718; recordedBy: J. Kim; individualCount: 2; sex: female; lifeStage: adult; occurrenceStatus: present; occurrenceID: 811C1827-27C0-51E2-9132-078552EC17AE; **Taxon:** scientificName: Valenzuela flavidus; kingdom: Animal; phylum: Arthropoda; class: Insecta; order: Psocodea; family: Caeciliusidae; genus: Valenzuela; taxonomicStatus: accepted; **Location:** country: South Korea; countryCode: KR; stateProvince: Gyeongsangnam-do; municipality: Jinju-si; locality: Gajwa-dong; verbatimLatitude: 35.09177; verbatimLongitude: 128.063607; geodeticDatum: WGS84; georeferenceProtocol: label; **Identification:** identifiedBy: J. Kim; dateIdentified: 2023; **Event:** samplingProtocol: hand collected; year: 2023; month: 7; day: 18; **Record Level:** type: specimen; language: en; rightsHolder: Gyeongsang National University; collectionID: GNU; collectionCode: Insects; basisOfRecord: PreservedSpecimen
- **Type status:** Other material. **Occurrence:** catalogNumber: coll#.JY-230718; recordedBy: J. Kim; individualCount: 2; sex: male; lifeStage: adult; occurrenceStatus: present; occurrenceID: EA16184C-F0DD-5710-8C37-99B81DF4FEB9; **Taxon:** scientificName: Valenzuela flavidus; kingdom: Animal; phylum: Arthropoda; class: Insecta; order: Psocodea; family: Caeciliusidae; genus: Valenzuela; taxonomicStatus: accepted; **Location:** country: South Korea; countryCode: KR; stateProvince: Gyeongsangnam-do; municipality: Jinju-si; locality: Gajwa-dong; verbatimLatitude: 35.09177; verbatimLongitude: 128.063607; geodeticDatum: WGS84; georeferenceProtocol: label; **Identification:** identifiedBy: J. Kim; dateIdentified: 2023; **Event:** samplingProtocol: hand collected; year: 2023; month: 7; day: 18; **Record Level:** type: specimen; language: en; rightsHolder: Gyeongsang National University; collectionID: GNU; collectionCode: Insects; basisOfRecord: PreservedSpecimen
- **Type status:** Other material. **Occurrence:** catalogNumber: coll#.JY-230805; recordedBy: J. Kim; individualCount: 2; sex: female; lifeStage: adult; occurrenceStatus: present; occurrenceID: DCF05A48-A20A-5A57-BD26-FD5EE0B1A713; **Taxon:** scientificName: Valenzuela flavidus; kingdom: Animal; phylum: Arthropoda; class: Insecta; order: Psocodea; family: Caeciliusidae; genus: Valenzuela; taxonomicStatus: accepted; **Location:** country: South Korea; countryCode: KR; stateProvince: Ulsan-si; municipality: Jung-gu; locality: Taehwa-dong; verbatimLatitude: 35.332664; verbatimLongitude: 129.174740; geodeticDatum: WGS84; georeferenceProtocol: label; **Identification:** identifiedBy: J. Kim; dateIdentified: 2023; **Event:** samplingProtocol: hand collected; year: 2023; month: 8; day: 5; **Record Level:** type: specimen; language: en; rightsHolder: Gyeongsang National University; collectionID: GNU; collectionCode: Insects; basisOfRecord: PreservedSpecimen
- **Type status:** Other material. **Occurrence:** catalogNumber: coll#.JY-230805; recordedBy: J. Kim; individualCount: 2; sex: male; lifeStage: adult; occurrenceStatus: present; occurrenceID: 4F1D0191-6145-5DFF-8676-5DF0A72B85E8; **Taxon:** scientificName: Valenzuela flavidus; kingdom: Animal; phylum: Arthropoda; class: Insecta; order: Psocodea; family: Caeciliusidae; genus: Valenzuela; taxonomicStatus: accepted; **Location:** country: South Korea; countryCode: KR; stateProvince: Ulsan-si; municipality: Jung-gu; locality: Taehwa-dong; verbatimLatitude: 35.332664; verbatimLongitude: 129.174740; geodeticDatum: WGS84; georeferenceProtocol: label; **Identification:** identifiedBy: J. Kim; dateIdentified: 2023; **Event:** samplingProtocol: hand collected; year: 2023; month: 8; day: 5; **Record Level:** type: specimen; language: en; rightsHolder: Gyeongsang National University; collectionID: GNU; collectionCode: Insects; basisOfRecord: PreservedSpecimen
- **Type status:** Other material. **Occurrence:** catalogNumber: coll#.JY-240722; recordedBy: J. Kim; individualCount: 2; sex: female; lifeStage: adult; occurrenceStatus: present; occurrenceID: D957EB36-C690-50C8-BCD9-23A87B350A74; **Taxon:** scientificName: Valenzuela flavidus; kingdom: Animal; phylum: Arthropoda; class: Insecta; order: Psocodea; family: Caeciliusidae; genus: Valenzuela; taxonomicStatus: accepted; **Location:** country: South Korea; countryCode: KR; stateProvince: Jeju-do; municipality: Seogwipo-si; locality: Daepo-dong; verbatimLatitude: 33.145962; verbatimLongitude: 126.255895; geodeticDatum: WGS84; georeferenceProtocol: label; **Identification:** identifiedBy: J. Kim; dateIdentified: 2024; **Event:** samplingProtocol: hand collected; year: 2024; month: 7; day: 22; **Record Level:** type: specimen; language: en; rightsHolder: Gyeongsang National University; collectionID: GNU; collectionCode: Insects; basisOfRecord: PreservedSpecimen
- **Type status:** Other material. **Occurrence:** catalogNumber: coll#.JY-240722; recordedBy: J. Kim; individualCount: 2; sex: male; lifeStage: adult; occurrenceStatus: present; occurrenceID: C0A4C2E3-6D47-56EA-8E07-96640E93BC6C; **Taxon:** scientificName: Valenzuela flavidus; kingdom: Animal; phylum: Arthropoda; class: Insecta; order: Psocodea; family: Caeciliusidae; genus: Valenzuela; taxonomicStatus: accepted; **Location:** country: South Korea; countryCode: KR; stateProvince: Jeju-do; municipality: Seogwipo-si; locality: Daepo-dong; verbatimLatitude: 33.145962; verbatimLongitude: 126.255895; geodeticDatum: WGS84; georeferenceProtocol: label; **Identification:** identifiedBy: J. Kim; dateIdentified: 2024; **Event:** samplingProtocol: hand collected; year: 2024; month: 7; day: 22; **Record Level:** type: specimen; language: en; rightsHolder: Gyeongsang National University; collectionID: GNU; collectionCode: Insects; basisOfRecord: PreservedSpecimen
- **Type status:** Other material. **Occurrence:** catalogNumber: coll#.JY-240814; recordedBy: J. Kim; individualCount: 2; sex: female; lifeStage: adult; occurrenceStatus: present; occurrenceID: 9FA13516-A328-5D77-BC38-5896E38030A4; **Taxon:** scientificName: Valenzuela flavidus; kingdom: Animal; phylum: Arthropoda; class: Insecta; order: Psocodea; family: Caeciliusidae; genus: Valenzuela; taxonomicStatus: accepted; **Location:** country: South Korea; countryCode: KR; stateProvince: Chungcheongnam-do; municipality: Taean-gun; locality: Seung-eon-ri; verbatimLatitude: 36.302862; verbatimLongitude: 126.213211; geodeticDatum: WGS84; georeferenceProtocol: label; **Identification:** identifiedBy: J. Kim; dateIdentified: 2024; **Event:** samplingProtocol: hand collected; year: 2024; month: 8; day: 14; **Record Level:** type: specimen; language: en; rightsHolder: Gyeongsang National University; collectionID: GNU; collectionCode: Insects; basisOfRecord: PreservedSpecimen
- **Type status:** Other material. **Occurrence:** catalogNumber: coll#.JY-240814; recordedBy: J. Kim; individualCount: 2; sex: male; lifeStage: adult; occurrenceStatus: present; occurrenceID: 5B3EC6AE-5CDA-5B42-A5F3-FA7568D721B2; **Taxon:** scientificName: Valenzuela flavidus; kingdom: Animal; phylum: Arthropoda; class: Insecta; order: Psocodea; family: Caeciliusidae; genus: Valenzuela; taxonomicStatus: accepted; **Location:** country: South Korea; countryCode: KR; stateProvince: Chungcheongnam-do; municipality: Taean-gun; locality: Seung-eon-ri; verbatimLatitude: 36.302862; verbatimLongitude: 126.213211; geodeticDatum: WGS84; georeferenceProtocol: label; **Identification:** identifiedBy: J. Kim; dateIdentified: 2024; **Event:** samplingProtocol: hand collected; year: 2024; month: 8; day: 14; **Record Level:** type: specimen; language: en; rightsHolder: Gyeongsang National University; collectionID: GNU; collectionCode: Insects; basisOfRecord: PreservedSpecimen
- **Type status:** Other material. **Occurrence:** catalogNumber: coll#.JY-250807; recordedBy: J. Kim; individualCount: 2; sex: female; lifeStage: adult; occurrenceStatus: present; occurrenceID: 403DD6DA-D37C-5DEA-96AB-C37633EA58B2; **Taxon:** scientificName: Valenzuela flavidus; kingdom: Animal; phylum: Arthropoda; class: Insecta; order: Psocodea; family: Caeciliusidae; genus: Valenzuela; taxonomicStatus: accepted; **Location:** country: South Korea; countryCode: KR; stateProvince: Gangwon-do; municipality: Wonju-si; locality: Heungeop-myeon; verbatimLatitude: 37.172256; verbatimLongitude: 127.545940; geodeticDatum: WGS84; georeferenceProtocol: label; **Identification:** identifiedBy: J. Kim; dateIdentified: 2025; **Event:** samplingProtocol: hand collected; year: 2025; month: 8; day: 7; **Record Level:** type: specimen; language: en; rightsHolder: Gyeongsang National University; collectionID: GNU; collectionCode: Insects; basisOfRecord: PreservedSpecimen
- **Type status:** Other material. **Occurrence:** catalogNumber: coll#.JY-250807; recordedBy: J. Kim; individualCount: 2; sex: male; lifeStage: adult; occurrenceStatus: present; occurrenceID: 50E435B5-4FE2-5687-9AA0-412DBD9F73A7; **Taxon:** scientificName: Valenzuela flavidus; kingdom: Animal; phylum: Arthropoda; class: Insecta; order: Psocodea; family: Caeciliusidae; genus: Valenzuela; taxonomicStatus: accepted; **Location:** country: South Korea; countryCode: KR; stateProvince: Gangwon-do; municipality: Wonju-si; locality: Heungeop-myeon; verbatimLatitude: 37.172256; verbatimLongitude: 127.545940; geodeticDatum: WGS84; georeferenceProtocol: label; **Identification:** identifiedBy: J. Kim; dateIdentified: 2025; **Event:** samplingProtocol: hand collected; year: 2025; month: 8; day: 7; **Record Level:** type: specimen; language: en; rightsHolder: Gyeongsang National University; collectionID: GNU; collectionCode: Insects; basisOfRecord: PreservedSpecimen

#### Description

**Figure 2.**
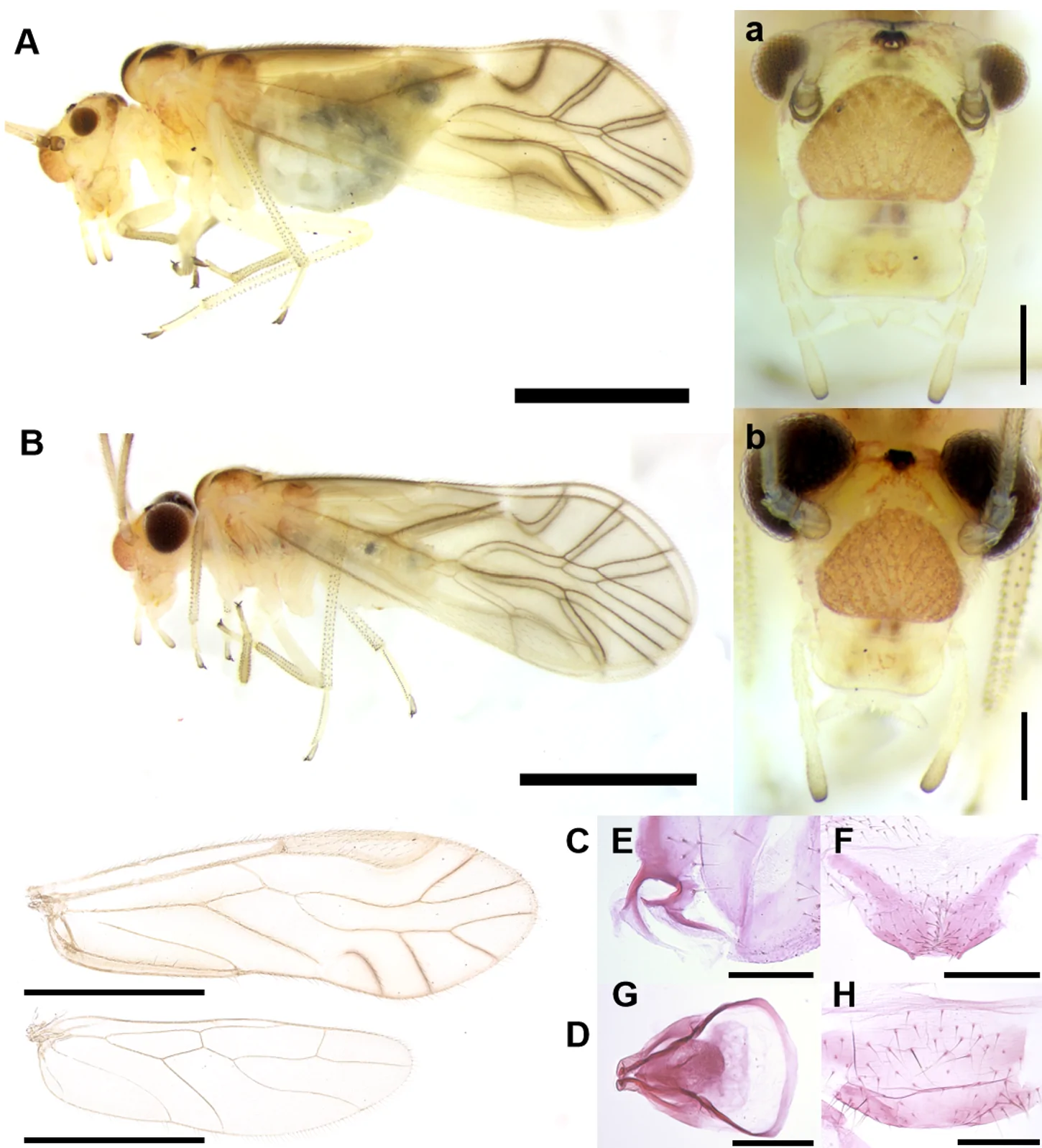
*Valenzuela flavidus* (Stephens, 1835). **A** female habitus; a, female head; **B** male habitus; b, male head; **C** fore-wing; **D** hind-wing; **E** gonapophyses; **F** subgenital plate; **G** phallosome; **H** hypandrium. Scale bars: A–D = 1 mm; a, b, F, H = 200 μm; E, G = 100 μm.

**Female**. Body generally bright yellow to creamy-yellow; meso- and metanotum medium brown (Fig. 2A). Head yellow, with a distinct brown median stripe along face. Compound eyes brown to reddish-brown. Ocellar field medium to dark brown; ecdysial line of vertex bordered with a pale brown band. Antennae yellow to pale brown; f1 usually paler than scape, pedicel and remaining flagellomeres (Fig. 2A). Fore-wing hyaline to very pale tawny yellow; wing markings mainly emphasised along veins, especially in distal half, where veins are bordered with reddish-brown. Additional pigmentation present in the posterior part of the anal area (Fig. 2C). Hind-wing hyaline; veins brown, except CuP and A, which are yellow (Fig. 2D). Subgenital plate with two distinct apical lobes (Fig. 2F). Gonapophyses with the basal part of dv acutely produced (Fig. 2E). **Male**. Similar to female in general colouration (Fig. 2B), but compound eyes relatively larger (Fig. 2B). Phallosome with parameres relatively thickened basally (Fig. 2G). Hypandrium simple and weakly sclerotised (Fig. 2H).

#### Distribution

Palaearctic Region (South Korea [in this study], Japan, Mongolia, Europe, Turkiye, former USSR), Nearctic Region (Canada, USA), Central America (Mexico, Nicaragua), Atlantic Islands (Azores, Bermuda Islands, Canary Islands, Madeira) (Lienhard and Smithers 2002).

#### Notes

This species is widely distributed throughout South Korea. It has been found on various tree species, including *Zelkova serrata*, *Quercus mongolica* and *Castanopsis sieboldii*.

#### Measurements (mm)

**Female** (n = 10). FW 2.85–3.20; HW 2.12–2.38; F 0.60–0.70; T 1.02–1.18; t1 0.34–0.40; t2 0.11–0.14; f1 0.45–0.53; f2 0.36–0.43. **Male** (n = 10). FW 2.70–3.05; HW 2.00–2.25; F 0.56–0.65; T 0.96–1.10; t1 0.31–0.37; t2 0.10–0.13; f1 0.47–0.56; f2 0.38–0.46.

## Identification Keys

### Identification key to the species of *Valenzuela* in South Korea

**Table:** 

1	Anal area of fore-wing distinctly pigmented	*Valenzuela flavidus*
–	Fore-wing generally darkened	2
2	Pterostigma brown, with a darker posterior margin	*Valenzuela badiostigma*
–	Pterostigma yellowish with black longitudinal stripes	*Valenzuela oyamai*

## Supplementary Material

XML Treatment for Valenzuela badiostigma

XML Treatment for Valenzuela flavidus
